# SEED-ML: A multi-parametric clinical dataset on male infertility for predictive modeling and AI research

**DOI:** 10.1016/j.dib.2026.112763

**Published:** 2026-04-09

**Authors:** N. Sánchez-Gómez, J.A. García-García, J. Navarro-Pando, M.J. Escalona-Cuaresma

**Affiliations:** a ES3 Group (Engineering and Science for Software Systems group), University of Seville, Avenida Reina Mercedes, s/n., Seville 41012, Spain; b Cátedra de Reproducción y Genética Humana del Instituto para el Estudio de la Biología de la Reproducción Humana (INEBIR), Seville, Spain; c Universidad Europea del Atlántico (UNEATLANTICO), Santander, Spain; d Fundación Universitaria Iberoamericana (FUNIBER), Seville, Spain; e San Juan de Dios Hospital, Sevilla, Spain

**Keywords:** Male infertility, Semen analysis, Clinical dataset, Artificial intelligence, Machine learning, Predictive modeling, Reproductive health

## Abstract

SEED-ML (Semen Examination and Evaluation Dataset for Machine Learning) is an openly available, multi-parametric clinical dataset specifically designed to support research in male infertility diagnostics and prediction. SEED-ML refers specifically to the dataset repository and its clinical structure, and not to a specific machine learning model or diagnostic method. In this sense, SEED-ML comprises records from 10,124 patients, including detailed semen analysis parameters (pre- and post-capacitation), morphological classifications, and clinical alterations. Infertility diagnosis is categorized into nine clinically relevant classes, ranging from normal fertility to complex multi-factor conditions such as oligoasthenoteratozoospermia. All data were anonymized and curated following strict ethical and privacy guidelines to ensure compliance with applicable medical data protection regulations. The dataset reflects real-world clinical distributions across nine diagnostic classes: Normozoospermia (62.68%), Oligoasthenoteratozoospermia (14.22%), Asthenozoospermia (11.66%), Teratozoospermia (6.71%), Oligozoospermia (1.90%), Asthenoteratozoospermia (1.38%), Oligoasthenozoospermia (0.96%), Oligoteratozoospermia (0.34%), and Azoospermia (0.16%). This detailed categorization provides a realistic clinical distribution for machine learning evaluation. SEED-ML offers a resource for developing and benchmarking machine learning models, enabling research in predictive analytics, decision support systems, and computational andrology. This dataset aims to facilitate interdisciplinary collaboration between clinicians, data scientists, and AI (artificial intelligence) researchers. The dataset is publicly available in Mendeley under a CC BY 4.0 license.

Specifications Table

**Table:** 

Subject	Health Sciences, Medical Sciences & Pharmacology
Specific subject area	Male infertility and reproductive medicine: clinical semen analysis and diagnostic test records
Type of data	CSV file, Raw, Filtered
Data collection	Data were collected from male patients at Assisted Reproduction Unit of the Institute for the Study of Human Reproductive Biology (INEBIR, Instituto para el Estudio de la Biología de la Reproducción Humana). Inclusion: males 20–64y undergoing fertility assessment or confirmed infertility. Exclusion: incomplete records or unrelated comorbidities. Data were pseudonymised, standardized, and consolidated to ensure a consistent format per observation. While multiple visits from the same patient are preserved, they are considered as independent clinical events to protect patient privacy and avoid the use of sensitive date-based identifiers.
Data source location	Institute for the Study of Human Reproductive Biology (INEBIR). Address: C. Radio Sevilla, 9, Casco Antiguo, 41001 Sevilla, Spain
Data accessibility	Repository name: Mendeley Direct URL to data [1]: https://data.mendeley.com/datasets/sc8rsz2vd7/1 DOI: 10.17632/sc8rsz2vd7.1
Related research article	None

## 1 Value of the Data

• Large-Scale Open Resource: With 10,124 patients records, the dataset provides one of the few openly available resources specifically designed for studying male infertility.
• Multi-class Diagnostic Precision: Unlike traditional binary classification models, SEED-ML’s nine diagnostic classes enable researchers to investigate the subtle physiological and computational boundaries between complex phenotypes, such as the OAT syndrome.
• Benchmarking and Clinical Validation: The dataset offers a resource for benchmarking machine learning models and facilitating independent clinical validation of diagnostic algorithms.
• Standardized Interoperability: The dataset's structure ensures compatibility with global clinical standards—specifically the WHO Laboratory Manual for the Examination and Processing of Human Semen (6th Edition) —ensuring that the parameters measured (e.g., concentration, motility, and morphology) are consistent with international clinical guidelines. This facilitates the integration of SEED-ML into data science workflows or its combination with other standardized reproductive health datasets.
• Predictive Weight of Rare Biomarkers: The inclusion of biochemical markers (citric acid, fructose) and DNA fragmentation data (SCD) allows to enable the evaluation of their predictive importance in large-scale machine learning models—markers that are missing in other public repositories.
• Optimization of Assisted Reproduction: By documenting both pre- and post-treatment states, the dataset provides a foundation for developing algorithms that predict sperm recovery rates and help in selecting the most effective capacitation technique (e.g., density gradients vs. swim-up) for specific patient profiles.

## 2 Background

Male infertility is a complex medical condition influenced by genetic, physiological, environmental, and lifestyle factors [2]. Despite its prevalence, publicly available datasets specifically dedicated to male infertility remain scarce, which limits the reproducibility of computational studies and the development of robust predictive models. For example, while existing repositories like the UCI Fertility Dataset [3] have been instrumental in early ML applications for reproductive health, they often suffer from limited sample sizes (N=100). Our dataset addresses these limitations by providing a massive cohort of 10,124 patients and 84 granular variables. Specifically, the inclusion of biochemical markers (citric acid, fructose) and DNA fragmentation (SCD) data—rarely found in public datasets like VISEM [4]—enables the development of more comprehensive models.

Most notably, our data captures the transition between pre- and post-treatment states, offering ML models to predict sperm recovery and therapeutic outcomes [2]. The motivation for compiling this dataset was to provide the research community with a structured and anonymized collection of clinical records that can support data-driven exploration of this health problem, using machine learning techniques.

The dataset was created within the context of applying machine learning and statistical methods to be applied into Assisted Reproductive Treatment (ART) contexts [5], with the aim of facilitating the evaluation of algorithms across standardized input features. By consolidating anonymized EHRs (Electronic Health Records) of 10,124 male patient cases into a consistent format, the dataset offers a reliable resource for benchmarking and methodological comparison. These patients are treated by medical professionals at the Assisted Reproduction Unit of INEBIR^1^.

This data paper complements original research, which has not yet been published and in which we will show how we have built and trained machine learning models to identify male infertility at an early stage. By bridging the gap between clinical andrology and data science, SEED-ML addresses several unresolved research questions. Specifically, it facilitates the study of how morphological abnormalities correlate with biochemical deficiencies and DNA integrity at a population level.

## 3 Data Description

This section aims to describe the columns and information in our data set in detail. The dataset contains a set of EHRs from male patients who are undergoing fertility treatment information. EHRs group for the consolidation of related diagnostic records with the standardization of diagnostic records to ensure a consistent format per observation, i.e., while multiple visits from the same patient are preserved, they are considered as independent clinical events to protect patient privacy and avoid the use of sensitive date-based identifiers. Each EHR contains values for the target categorical variable (which is titled «diagnostic») and 84 medical variables.

Moreover, male infertility can be classified according to alterations in sperm concentration, motility, and morphology. In this sense, different clinical typologies are recognized to express the classes of the target categorical variable (cf., Table 1) [6]. The distribution of diagnostic classes in SEED-ML reflects the natural prevalence of male infertility phenotypes in a specialized clinical setting. Normozoospermia (NO) is the most frequent category, accounting for 62.68% (n = 6,346) of the records. Among pathological conditions, the OAT syndrome (14.22%) and Asthenozoospermia (11.66%) are the most prevalent, providing a robust basis for training models on complex motility and multi-parametric disorders. Conversely, severe or rare conditions such as Oligotheratozoospermia (0.34%) and Azoospermia (0.16%) represent a significant minority. This inherent class imbalance mirrors real-world clinical practice in reproductive units.

**Table 1:** Distribution of clinical diagnostic categories in the SEED-ML dataset (N = 10,124).

Classes and Meaning	Count (N); Percentage (%)
No: It refers to the patient not suffering from male infertility.	6346; 62.68%
Azoospermia: It refers to the complete absence of spermatozoa in the ejaculate.	16; 0.16%
Oligozoospermia: It designates a reduced sperm concentration below the reference thresholds defined by the World Health Organization (WHO).	192; 1.90%
Asthenozoospermia: It reflects alterations in motility.	1180; 11.66%
Theratozoospermia: It reflects morphological abnormalities predominate.	679; 6.71%
Oligoasthenozoospermia: It corresponds to reduced concentration together with impaired motility.	97; 0.96%
Oligotheratozoospermia: It combines low concentration with abnormal morphology.	34; 0.34%
Asthenotheratozoospermia: It reflects the simultaneous alteration of motility and morphology.	140; 1.38%
Oligoasthenotheratozoospermia: It is the OAT syndrome and is the most severe clinical presentation, in which concentration, motility, and morphology are all affected.	1440; 14.22%
Total	10,124; 100%

The remaining variables in the dataset correspond to parameters derived from semen analysis (seminogram) [7], which is the standard clinical test for assessing male fertility. This analysis quantitatively and qualitatively characterizes semen samples through the measurement of parameters such as sperm concentration, motility, morphology, and vitality, which serve as key indicators of fertilizing potential. In the context of assisted reproduction procedures, semen samples are analyzed in two distinct phases: pre-treatment and post-treatment. Pre-treatment refers to the baseline evaluation of the semen sample in its original state, following standardized protocols for macroscopic and microscopic examination [6]. Conversely, post-treatment corresponds to the sample assessment after processing through sperm selection or capacitation techniques, such as density gradient centrifugation or swim-up methods [6]. These laboratory procedures are designed to remove seminal plasma, debris, and non-viable cells, thereby isolating a population of spermatozoa with superior motility and higher fertilization potential [2].

Below, the list of the variables in the dataset is presented^2^, describing the variable name, its data type (i.e., String, Integer, Boolean, and Decimal), and its description.

1. Diagnostic [String]: Diagnostic of male infertility (Target Variable). Recorded values are described in Table 1. These labels are assigned by specialist clinicians based on the WHO 6th Edition criteria [6]. While some classes are threshold-defined, the dataset includes complex multi-factor phenotypes (e.g., OAT) where diagnostic value emerges from the non-linear interaction of multiple parameters. Researchers are encouraged to use this label in conjunction with the modeling guidelines provided in Section 4.4 to avoid circular reasoning.
2. sample_agglutination [String]: Sample agglutination (General information). Recorded values: –, No agglutination observed (normal); +, Mild agglutination (<10% of sperm affected); ++, Moderate agglutination (10–50%); +++, Intense agglutination (>50%); ++++, Massive or complete agglutination.
3. sample_anormal_total_post [Integer]: Abnormal totals - post treatment
4. sample_anormal_total_pre [Integer]: Abnormal totals - pre-treatment
5. sample_appearance [String]: Sample appearance (General information). Recorded values: Normal, Yellowish, White, Translucent
6. sample_bodies_gelatinous [Boolean]: Gelatinous bodies (General information)
7. sample_cells_germinal [Decimal]: Germinal cells (Treatment)
8. sample_cells_round [Decimal]: Round cells (General information)
9. sample_citric [Decimal]: Citric acid (Biochemical test)
10. sample_concentration_initial [Decimal]: Initial concentration (General information)
11. sample_drops [Integer]: Drops (Treatment)
12. sample_fructose [Integer]: Fructose (Biochemical test)
13. sample_heads_amorphous_post [Integer]: Amorphous (sperm heads) - post treatment
14. sample_heads_amorphous_pre [Integer]: Amorphous (sperm heads) - pre-treatment
15. sample_heads_combined_post [Integer]: Combined (sperm heads) - post treatment
16. sample_heads_combined_pre [Integer]: Combined (sperm heads) - pre-treatment
17. sample_heads_double_post [Integer]: Double head (sperm heads) - post treatment
18. sample_heads_double_pre [Integer]: Double head (sperm heads) - pre-treatment
19. sample_heads_elongated_post [Integer]: Elongated (sperm heads) - post treatment
20. sample_heads_elongated_pre [Integer]: Elongated (sperm heads) - pre-treatment
21. sample_heads_macrocephalus_post [Integer]: Macrocephalic (sperm heads) - post treatment
22. sample_heads_macrocephalus_pre [Integer]: Macrocephalic (sperm heads) - pre-treatment
23. sample_heads_microcephalus_post [Integer]: Microcephalic (sperm heads) - post treatment
24. sample_heads_microcephalus_pre [Integer]: Microcephalic (sperm heads) - pre-treatment
25. sample_heads_piriform_post [Integer]: Pyriform (sperm heads) - post treatment
26. sample_heads_piriform_pre [Integer]: Pyriform (sperm heads) - pre-treatment
27. sample_heads_round_post [Integer]: Round (sperm heads) - post treatment
28. sample_heads_round_pre [Integer]: Round (sperm heads) - pre-treatment
29. sample_heads_small_acrosome_post [Integer]: Small acrosome (sperm heads) - post treatment
30. sample_heads_small_acrosome_pre [Integer]: Small acrosome (sperm heads) - pre-treatment
31. sample_heads_total_post [Integer]: Total (sperm heads) - post treatment
32. sample_heads_total_pre [Integer]: Total (sperm heads) - pre-treatment
33. sample_heads_vacuole_post [Integer]: Vacuoles (sperm heads) - post treatment
34. sample_heads_vacuole_pre [Integer]: Vacuoles (sperm heads) - pre-treatment
35. sample_leukocytes [Integer]: Leukocytes (General information)
36. sample_liquefaction [String]: Sample liquefaction (General information). Recorded values: Complete, Incomplete
37. sample_morpho_kruger [Integer]: Kruger morphology (General information)
38. sample_morpho_normal [Decimal]: Normal morphology (General information)
39. sample_morphology_normal_post [Integer]: Normal morphology - post treatment
40. sample_morphology_normal_pre [Integer]: Normal morphology - pre-treatment
41. sample_necks_bent_post [Integer]: Bent neck (sperm neck) - post treatment
42. sample_necks_bent_pre [Integer]: Bent neck (sperm neck) - pre-treatment
43. sample_necks_combined_post [Integer]: Combined neck (sperm neck) - post treatment
44. sample_necks_combined_pre [Integer]: Combined neck (sperm neck) - pre-treatment
45. sample_necks_ins_asymmetric_post [Integer]: Asymmetric neck insertion (sperm neck) - post treatment
46. sample_necks_ins_asymmetric_pre [Integer]: Asymmetric neck insertion (sperm neck) - pre-treatment
47. sample_necks_tails_total_post [Integer]: Total neck (sperm neck) - post treatment
48. sample_necks_tails_total_pre [Integer]: Total neck (sperm neck) - pre-treatment
49. sample_necks_thick_post [Integer]: Thick neck (sperm neck) - post treatment
50. sample_necks_thick_pre [Integer]: Thick neck (sperm neck) - pre-treatment
51. sample_necks_thin_post [Integer]: Thin neck (sperm neck) - post treatment
52. sample_necks_thin_pre [Integer]: Thin neck (sperm neck) - pre-treatment
53. sample_num_prog_mob_total [Decimal]: Total number of progressive motile sperm (General information)
54. sample_num_spz_counted_post [Integer]: Number of sperm counted - post treatment
55. sample_num_spz_counted_pre [Integer]: Number of sperm counted - pre-treatment
56. sample_num_spz_normal_post [Integer]: Number of normal sperm - post treatment
57. sample_num_spz_normal_pre [Integer]: Number of normal sperm - pre-treatment
58. sample_ph [Decimal]: Degree of acidity or alkalinity (pH) (General information)
59. sample_production_total [Decimal]: Total production (General information)
60. sample_production_total_final [Decimal]: Final total production - post treatment
61. sample_recovery_technique [String]: Technique (Treatment). Recorded values: Gradient, Concentrated Wash, Standard Swim
62. sample_red_blood_cells [String]: Red blood cells (General information). Recorded values: Complete, Incomplete
63. sample_scd [Integer]: SCD - Sperm Chromatin Dispersion Test - (General information)
64. sample_spermatids [Integer]: Spermatids (Treatment)
65. sample_spermatocyte1 [Integer]: Spermatocyte 1 (Treatment)
66. sample_spermatocyte2 [Integer]: Spermatocyte 2 (Treatment)
67. sample_spermatogoniums [Integer]: Spermatogonia (Treatment)
68. sample_spz_swollen [Integer]: Swollen sperm (HOST test)
69. sample_state [String]: Sample state (General information). Recorded values: Complete Sample, Medium Loss
70. sample_survival_test [Integer]: Survival test (General information)
71. sample_tails_broken_post [Integer]: Broken tail (sperm tail) - post treatment
72. sample_tails_broken_pre [Integer]: Broken tail (sperm tail) - pre-treatment
73. sample_tails_combined_post [Integer]: Combined tail (sperm tail) - post treatment
74. sample_tails_combined_pre [Integer]: Combined tail (sperm tail) - pre-treatment
75. sample_tails_multiple_post [Integer]: Multiple tails (sperm tail) - post treatment
76. sample_tails_multiple_pre [Integer]: Multiple tails (sperm tail) - pre-treatment
77. sample_tails_rolled_post [Integer]: Rolled tail (sperm tail) - post treatment
78. sample_tails_rolled_pre [Integer]: Rolled tail (sperm tail) - pre-treatment
79. sample_tails_short_post [Integer]: Short tail (sperm tail) - post treatment
80. sample_tails_short_pre [Integer]: Short tail (sperm tail) - pre-treatment
81. sample_teratozoospermia_index [Decimal]: Teratozoospermia index (Treatment)
82. sample_type_treat_recovery [Decimal]: Type (Treatment)
83. sample_viscosity [String]: Sample viscosity (General information). Recorded values: Normal, Increased
84. sample_vitality [Integer]: Vitality (General information)
85. sample_vol_initial [Decimal]: Initial volume (General information)

To improve feature organization, the variables were grouped into five functional clusters, which represent the sequential stages of a comprehensive semen analysis and laboratory processing. The first two groups, (1) Macroscopic & Physical Evaluation and (2) Microscopic & Vitality Analysis, capture the baseline fluid properties and sperm quantification. The (3) Detailed Morphological Defects (Pre) cluster provides high-granularity data on 32 specific structural anomalies observed before treatment. For more advanced physiological insights, the (4) Specialized Biomarkers group includes molecular and biochemical indicators such as DNA fragmentation and citric acid levels. Finally, the (5) Laboratory Processing Outcomes (Post) group contains all parameters measured after sperm capacitation, serving as the primary targets for prognostic modeling. This categorization, detailed in Table 2, provides a systematic roadmap for feature selection and domain-specific analysis.

**Table 2:** Functional grouping and full variable list for macroscopic and microscopic categories.

Cluster	Variables Included	Meaning	#
1. Macroscopic & Physical	sample_state, sample_appearance, sample_agglutination, sample_viscosity, sample_liquefaction, sample_red_blood_cells, sample_ph, sample_vol_initial, sample_bodies_gelatinous	Describes the physical properties of the seminal plasma (volume, acidity, consistency).	9
2. Microscopic & Vitality Analysis	sample_concentration_initial, sample_vitality, sample_cells_round, sample_leukocytes, sample_survival_test, sample_production_total, sample_num_prog_mob_total, sample_morpho_normal, sample_morpho_kruger, sample_num_spz_counted_pre, sample_num_spz_normal_pre, sample_morphology_normal_pre	Quantitative measurements of sperm count, survival rate, and presence of non-sperm cells.	12
3. Detailed Morphology (Pre)	32 variables with Suffix _pre (e..g., heads_amorphous_pre, necks_thick_pre, tails_broken_pre, etc.)	Exhaustive classification of structural defects in the head, neck, and tail before treatment.	32
4. Specialized Biomarkers	sample_fructose, sample_citric_acid, sample_scd	Molecular and biochemical indicators of glandular function and DNA integrity.	3
5. Laboratory Processing Outcomes (Post)	28 variables with Suffix _final or _post (e..g., concentration_final, production_total_final, morpho_normal_final)	Parameters measured after laboratory processing (capacitation) to evaluate treatment success.	28

Finally, to ensure the correct application of the dataset in AI research, two primary modeling objectives are defined: diagnostic modeling, and laboratory outcome prediction. On the one hand, with regard to diagnostic modeling, researchers should use pre-treatment variables (e.g., initial concentration, pH, volume) to predict the 'diagnostic' target variable. Using post-treatment variables for this task is discouraged as it may introduce data leakage or clinical inconsistencies, given that the diagnosis is traditionally established based on the baseline semen profile. On the other hand, with regard to laboratory outcome prediction, researchers may use the transition from pre- to post-treatment states to model laboratory efficiency. In this scenario, pre-treatment features act as inputs, while post-treatment parameters (such as 'sample_production_total_final') serve as the regression or classification targets to predict the success of sperm capacitation techniques.

## 4 Experimental Design, Materials and Methods

### 4.1 Study setting

This study was carried out in the Assisted Reproduction Unit of INEBIR, a reference center in reproductive medicine in Andalusia (Spain). It performs a large number of assisted reproduction treatments annually, which provides a clinically consistent patient cohort. The data presented in this paper were acquired from INEBIR’S EHR system (i.e., iMedea [8]). This system structurally stores detailed patient information, including demographic and family history, anamnesis, results of diagnostic tests, semen analyses, and records of fertility treatments, among other medical examinations. The standardized structure of iMedea guarantees the uniform registration of patient data, which facilitates both the clinical follow-up of patients and the secondary use of data for research purposes. The data collection started in 2015 and covers a continuous period of clinical activity, providing a large dataset with longitudinal consistency. The dataset includes male patients with a wide spectrum of reproductive conditions, ranging from normozoospermic individuals to those diagnosed with asthenospermia, teratospermia, oligospermia, and oligoasthenoteratospermia (OAT). This variety reflects common clinical presentations within the study setting. All data are shared under the CC BY 4.0 license, unless otherwise stated.

### 4.2 Study participants, sample and data collection

All data included in the dataset were originally collected during routine clinical practice and fertility assessments conducted at the Assisted Reproduction Unit of Inebir, under the supervision of experienced healthcare professionals specialized in reproductive medicine and urology. This clinical setting ensures both the quality and reliability of the medical records, as all information was obtained following standardized diagnostic protocols applied in daily medical practice. The initial sample consisted of diagnostic test results and medical records from 15,264 male patients who attended the clinic between 2015 and 2025. To ensure the internal validity and representativeness of the dataset, a strict selection process was applied based on predefined inclusion and exclusion criteria:

• Inclusion criteria: male patients aged 20 to 64 years, either undergoing assisted reproduction treatments or with a confirmed medical diagnosis of infertility.
• Exclusion criteria: patients outside the specified age range, with incomplete or inconsistent medical records, or with unrelated health conditions (e.g., systemic illnesses or treatments) that could interfere with reproductive capacity.

Following this procedure, a total of 10,124 medical histories were retained for analysis. This final sample size provides a sufficiently robust basis for clinical and epidemiological research, covering a wide variety of reproductive conditions and ensuring a representative cross-section of the male infertility population treated at Inebir. Before data processing, it was verified that all patients had provided informed consent allowing the use of their anonymised medical data for research purposes.

The study was conducted in accordance with the Declaration of Helsinki and the EU Data Protection Directive (Directive 95/46/EC) [9] and GDPR (Regulation EU 2016/679) [10]. In this sense, to ensure patient privacy and confidentiality, a rigorous pseudonymisation procedure was applied. Direct access to identifiable patient information was restricted exclusively to clinical researchers at Inebir. All external collaborators worked only with pseudonymised datasets. The pseudonymisation process combined several complementary techniques: (*i*) suppression of sensitive data, that means elimination of free-text fields and direct identifiers (e.g., episode IDs, medical record IDs, registration dates); (*ii*) recalculation for the transformation of date of birth into age in years; (*iii*) replacement and obfuscation, that means substitution of relational database identifiers with randomly generated unique identifiers; (*iv*) disaggregated information grouping for the consolidation of related diagnostic records (e.g., semenogram data) with the standardization of diagnostic records to ensure a consistent format per observation; (*v*) while multiple visits from the same patient are preserved, they are considered as independent clinical events to protect patient privacy and avoid the use of sensitive date-based identifiers; and (*vi*) data destruction, that means permanent deletion of temporary files containing raw datasets, retaining only the processed pseudonymised dataset.

This methodology ensured that the dataset was both clinically reliable—as it originated from standardized diagnostic protocols supervised by qualified specialists—and ethically robust, by complying with the highest standards of data protection and patient privacy.

### 4.3 Data preprocessing and quality assurance

After verifying the anonymisation of the data, a rigorous data preprocessing and quality assurance pipeline was implemented to ensure the completeness, consistency, and validity of the SEED-ML dataset, making it suitable for advanced machine learning applications. The entire process was performed using Python (version 3.9) with the Pandas and NumPy libraries. The key stages of this pipeline are detailed below.

#### 4.3.1 Data cleaning and consistency checks

Duplicate records were detected and removed, inconsistent formats were corrected, and categorical variables were standardized according to predefined coding schemes. Continuous variables were reviewed for outliers, and extreme or implausible values were flagged for verification. This stage involved:

• Duplicate Entry Removal: Any identified duplicates were removed to prevent biased model training and ensure data integrity. We scanned the dataset for duplicate records based on a multi-stage de-duplication process. Records were cross-referenced using a composite key consisting of the Unique Patient Identifier and the Laboratory Timestamp. When multiple entries for the same patient and date were found, we compared the readings; if the data were identical, the redundant record was removed. In cases of partial data overlap, the entry with the highest degree of completeness (lowest count of missing or 'False' values) was prioritized. Distinct clinical visits from the same patient on different dates were preserved as independent observations.
• Format Standardization: We standardized the format for all variables. For example, dates were converted to a consistent YYYY-MM-DD format, and text-based entries were normalized to a uniform case (e.g., lowercase) to avoid misinterpretation. In the format of date fields or timestamps, the standardization was carried out during the preprocessing stage of the original data source before obtaining the final dataset
• Categorical Variable Encoding: Categorical variables (such as infertility diagnosis, sample agglutination, or sample appearance, among others) were verified for correct spelling and standardized values.
• Outlier Detection: We performed a preliminary analysis to identify and manage statistical outliers in key numerical variables. Specifically, statistical outliers for numerical variables (e.g., age, sperm concentration, and motility) were identified using the Interquartile Range (IQR) method. Specifically, values falling outside the range (cf., Equation 1) were flagged as potential outliers. Implausible values (e.g., negative volumes or concentrations exceeding biological limits) were removed, while extreme but clinically possible values were retained to preserve the dataset's real-world variability. To distinguish between measurement errors and rare but biologically plausible extreme values, all flagged entries were cross-referenced with clinical reference ranges established by the WHO [6]. In this review process, the work of Inebir's healthcare professionals was fundamental and essential in identifying clinically abnormal values. For instance, while a sperm concentration of 0 cells/ml is a statistical outlier, it represents a valid clinical condition (azoospermia) and was retained. Conversely, values such as a pH above 10 or negative volumes were identified as technical outliers and excluded as they are biologically implausible according to WHO standards [6]. Finally, when an outlier is identified, its value is excluded by setting it to False.
  [Q1−1.5×IQR,Q3+1.5×IQR]

Equation 1. Range for identifying outlier values using the Interquartile Range (IQR) method

#### 4.3.2 Handling of missing data

Patterns of missingness were systematically evaluated across the different clinical data domains (e.g., patient history, laboratory results, and treatment procedures). Records with critical missing clinical information, such as absent semen analysis or key diagnostic results, were excluded during the eligibility phase. Given the clinical nature of the data, some variables had naturally high rates of missing values (e.g., parameters within the treatment domain for patients who did not undergo specific laboratory procedures). For non-critical missing variables, appropriate imputation strategies were considered to maintain data integrity while avoiding bias.

• Exclusion Criteria: Patients with critical missing clinical information that was essential for the main classification task (e.g., lack of a confirmed infertility diagnosis) were excluded during the initial eligibility phase. This decision was based on the premise that these records would not provide sufficient information for training a robust model.
• Imputation Strategy: For non-critical variables, where missingness was random and at an acceptable rate (e.g., < 5%), we chose not to perform data imputation to preserve the integrity of the original observations. We adopted a complete-case analysis approach for the final dataset, where only records with all necessary variables were included. This ensures that any subsequent analysis is performed on a complete and high-quality subset, minimizing the risk of introducing synthetic data that could skew the results.

We recognize that complete-case analysis may introduce selection bias if the missing data is informative (Missing Not At Random). In our dataset, missingness in the treatment domain is structural, as it corresponds to patients who did not proceed to sperm capacitation. For non-structural variables with missingness <5%, we prioritized the preservation of original clinical observations over synthetic imputation to avoid the introduction of potential biases or artifacts in a benchmarking resource. Nevertheless, we provide the raw data to allow researchers to implement alternative strategies, such as Multiple Imputation or Likelihood-based methods, depending on their specific research objectives.

Finally, it is important to note that SEED-ML does not include synthetic data or imputed values. All missing records are preserved in their original state to allow researchers to implement their own data-cleaning and imputation protocols. This approach ensures that any subsequent modeling reflects the true clinical variability and challenges of real-world medical data without pre-analytical bias

#### 4.3.3 Normalization and validation controls

In terms of standardisation and coding, laboratory measurements (e.g., sperm concentration, motility, hormone levels) were converted to standardised units to ensure comparability. Categorical variables were coded consistently across all records, allowing for harmonisation of data collected over several years and ensuring uniform representation across the dataset. Furthermore, with regard to validation checks, two researchers independently verified random samples of pseudonymised records to ensure consistency between the raw clinical data and the processed dataset. Cross-validation between the different data domains—including diagnostic background, primary semen analysis, and laboratory processing outcomes— ensured accuracy during the consolidation of the final dataset.

#### 4.3.4 Management of redundancy and feature prioritization

With 84 clinical variables, the SEED-ML dataset presents a high-dimensional space that requires careful management to ensure model interpretability and avoid the ``curse of dimensionality.'' While some structural redundancy exists (e.g., total sperm counts derived from volume and concentration), these features are preserved to support diverse engineering strategies. To guide researchers, we propose a three-tier prioritization framework based on clinical relevance and information density:

• Priority 1: WHO Core Diagnostic Drivers (Clusters 1 and 2; cf., Table 2). This tier includes primary parameters such as concentration, total motility, and morphology (Kruger). These variables are the reference for clinical diagnosis. Due to their high signal-to-noise ratio, they are recommended for establishing baseline model performance and ensuring immediate clinical validity.
• Priority 2: Deep Phenotyping and Research Markers (Clusters 3 and 4; cf., Table 2). This tier comprises 32 granular morphological defects and specialized biomarkers (e.g., Fructose, SCD). Although these features may exhibit higher multicollinearity, they are essential for Discovering latent biological patterns. We specifically recommend using Explainable AI (XAI) techniques, such as SHAP or LIME, on this tier to identify which specific structural anomalies (e.g., midpiece defects vs. nuclear vacuoles) most significantly impact fertility beyond standard metrics.
• Priority 3: Prognostic and Outcome Variables (Cluster 5; cf., Table 2). This tier contains variables measured after laboratory processing (capacitation). These should primarily be considered as targets for prognostic modeling or used in longitudinal studies to evaluate a sample’s ``functional recovery.'' Using these as input features for diagnostic tasks is discouraged to prevent data leakage, as they represent the clinical outcome of the sample’s laboratory preparation.

This hierarchical approach allows researchers to reduce the 84-variable space into manageable, interpretable domains, facilitating the development of transparent and clinically actionable AI tools.

### 4.4 Potential research applications and modeling considerations

To facilitate comparative research and ensure reproducibility, we propose the following benchmarking framework for the SEED-ML dataset:

1. Primary Task: Multi-class Diagnostic Classification. The core challenge involves predicting the nine clinical categories defined in Table 1. Given the clinical nature of the data, researchers should prioritize Macro-F1 Score and Balanced Accuracy as evaluation metrics to account for potential class imbalances.
2. Secondary Task: Post-treatment Outcome Prediction. Using pre-treatment variables as inputs to predict sperm recovery potential (e.g., final total production) after capacitation. This task is essential for developing decision-support systems in assisted reproduction.
3. Baseline Consideration: The provided raw and cleaned versions of the 84 variables serve as the starting point for feature engineering. Baseline models should ideally report performance using the 10,124 patient records to maintain comparability across future studies.
4. Mitigating Circular Reasoning: Given that some diagnostic labels are derived from standard WHO thresholds (e.g., concentration for Oligozoospermia), researchers are encouraged to design experiments that avoid trivial classification. We recommend carrying out a feature-restricted modeling and complex phenotype analysis. On the one hand, attempting to predict clinical classes using only biochemical markers (citric acid, fructose) and DNA fragmentation (SCD), excluding the primary defining parameters (concentration, motility, morphology). This approach allows for the discovery of latent correlations between molecular integrity and macroscopic semen quality. . On the other hand, focusing on the differentiation between multi-factor syndromes (e.g., Oligoasthenoteratozoospermia vs. Oligotheratozoospermia), where simple threshold-based rules are less effective due to the synergistic nature of the parameters.
5. Handling Minority Classes: Researchers should note the 'long tail' distribution of certain phenotypes. For rare classes such as Azoospermia (n=16) or Oligoteratozoospermia (n=34), we recommend the application of data augmentation techniques, such as SMOTE (Synthetic Minority Over-sampling Technique), or the use of cost-sensitive learning (weighted loss functions). These strategies are essential to ensure that predictive models do not ignore clinically critical but statistically infrequent conditions.

## Limitations

A relevant limitation of the dataset is that the cohort comes from a single assisted reproduction center (INEBIR, Seville), accredited as a center of excellence by the European Society of Human Reproduction and Embryology (ESHRE). This circumstance offers methodological advantages, as it ensures the application of standardized diagnostic protocols, uniform criteria for data collection, and a high-quality clinical framework. However, it also introduces limitations in terms of external validity. Patients attended in a single center may present a specific sociodemographic, cultural, and epidemiological profile, including specific ethnic backgrounds and lifestyle factors of the local healthcare system [11]. These particularities may affect the prevalence of certain infertility phenotypes, limiting the direct extrapolation of predictive models to diverse global populations. Consequently, predictive models derived from this resource should be interpreted with caution and within this restriction.

Although the dataset constitutes a standardized resource generated in an internationally recognized center, validation in multicentric cohorts, spanning different healthcare systems and diverse ethnic and socioeconomic groups, will be necessary to ensure the global applicability and fairness of AI-driven diagnostic tools in andrology. Furthermore, while pseudonymisation ensured data privacy, the removal of certain identifiers (e.g., detailed markers or free-text notes) limited the possibility of longitudinal follow-up and deeper analysis. Together, these factors should be considered when interpreting the findings.

Finally, another limitation is the absence of extra-testicular variables, such as genetic markers, or lifestyle and environmental exposure data (e.g., smoking habits, BMI, occupational heat exposure). While these factors are critical for understanding the etiology of infertility, SEED-ML is intentionally focused on the clinical semen analysis and the laboratory transition from pre- to post-treatment states. Consequently, the dataset is optimized for diagnostic and prognostic modeling within the laboratory setting, and researchers should be aware that its predictive power may be enhanced in the future by integrating these broader contextual variables from other sources.

## Ethics Statement

This study involved human participants and was conducted in accordance with the ethical principles set forth in the Declaration of Helsinki. Approval was granted by the Ethics Committee of the European University of the Atlantic, as recorded in Act number 78, under registration number CEI-40/2023, dated 05 October 2023. Prior to participation, informed consent was obtained from all subjects. These measures ensure full compliance with relevant legal and ethical standards concerning the use of patient data.

## CRediT Author Statement

**N. Sánchez-Gómez:** Data curation, Writing, Original draft preparation; **J.A. García-García:** Conceptualization; Methodology; Formal analysis; Writing (original draft, review and editing); **J. Navarro-Pando:** Formal analysis; Investigation; Validation (medical review of the manuscript); **MJ Escalona-Cuaresma:** Conceptualization; Investigation; Writing (review and editing).

## Data Availability

Mendeley DataSEED-ML: A Multi-Parametric Clinical Dataset on Male Infertility for Predictive Modeling and AI Research. (Original data).

## Footnotes

1 INEBIR (Institute for the Study of Human Reproductive Biology; Instituto para el Estudio de la Biología de la Reproducción Humana) is one of the leading healthcare entities in reproductive medicine in Andalusia (Spain), with more than 1200 ART cycles performed annually and more than 5500 ART cycles performed in total since 2020. Website: https://inebir.com/en/.

2 It is important to mention an observation about the presence of ‘False’ values in the dataset. As we have mentioned in this paper, Inebir uses iMedea [4] as its Health Information System (HIS). The architecture of iMedea is based on the Odoo framework. In Odoo's ORM, any field (regardless of its type: integer, decimal, or string) that has not been filled in on the clinical form is automatically stored and exported as a ‘false’ value by default, rather than a NULL or NaN. To ensure data integrity, we have classified “false” entries as follows: (i) In the 83 non-Boolean variables, 'False' denotes a missing data (NaN) due to the system's default export configuration; (ii) In the Boolean variable (e.g., ‘sample_bodies_gelatinous’), ‘False’ is a valid logical value indicating the absence of the trait.
